# Dezocine for Preventing Postoperative Pain: A Meta-Analysis of Randomized Controlled Trials

**DOI:** 10.1371/journal.pone.0136091

**Published:** 2015-08-19

**Authors:** Xuelong Zhou, Chenjing Zhang, Min Wang, LiNa Yu, M. Yan

**Affiliations:** 1 Department of Anesthesiology, The Second Affiliated Hospital, School of Medicine, Zhejiang University, Hangzhou, China; 2 Department of Gastroenterology, The Second Affiliated Hospital, School of Medicine, Zhejiang University, Hangzhou, China; 3 Jiangsu Key Laboratory of Anesthesiology, Xuzhou Medical College, Xuzhou, China; Scientific Inst. S. Raffaele Hosp., ITALY

## Abstract

**Background:**

Dezocine is considered to be an alternative medication for managing postoperative pain. The aim of this study was to assess the efficacy and safety of this drug in this regard.

**Methods:**

Medline, EMBASE and the Cochrane Central Register of Control Trials (CENTRAL) were searched to identify all randomized controlled trials (RCTs) that compare dezocine with placebo or dezocine with morphine on postoperative pain. The data were extracted and pooled using Mantel-Haenszel random effects model. Heterogeneity was tested using the *I* ^2^ statistic with values >50% and Chi^2^ test with P ≤ 0.05 indicating obvious heterogeneity between the studies.

**Results:**

Seven trials evaluating 665 patients were included. The number of patients with at least 50% pain relief was increased (N = 234; RR 3.04, 95% CI 2.27 to 4.08) and physician (N = 465; RR 2.84, 95% CI 1.66 to 4.84) and patient satisfaction (N = 390; RR 2.81, 95% CI 1.85 to 4.26) were improved following the administration of dezocine compared with the placebo. The effects of dezocine were similar to those of morphine in terms of the number of patients reporting at least 50% pain relief within 2–6 h after surgery (N = 235; RR 1.29, 95% CI 1.15 to 1.46) and physician (N = 234; RR 1.18, 95% CI 0.93 to 1.49) and patient (N = 158; RR 1.33, 95% CI 0.93 to 1.92) satisfaction. While, the number of patients with at least 50% pain relief within 0–1 h after surgery increased following dezocine compared with morphine treatment (N = 79; RR 1.45, 95% CI 1.18 to 1.77). There was no difference in the incidence of postoperative nausea and vomiting (PONV) following dezocine treatment compared with the placebo (N = 391; RR 1.06, 95% CI 0.42 to 2.68) or morphine treatment (N = 235; RR 0.65, 95% CI 0.14 to 2.93).

**Conclusion:**

Dezocine is a promising analgesic for preventing postoperative pain, but further studies are required to evaluate its safety.

## Introduction

Postoperative pain not only causes physical discomfort and psychological harm, it also induces differing degrees of endocrine hormone changes. It causes cardiovascular and pulmonary complications, prolongs hospital stays, and increases medical expenses [1–3]. Adequate postoperative analgesia reduces patient pain and corrects the abnormal endocrine response after surgery, thus promoting patient recovery [3–5].

Opioids are commonly used to manage postoperative pain. However, their analgesic effects are offset by undesired adverse effects, including respiratory depression, drug addiction, and nausea and vomiting [3,5]. Dezocine, a mixed agonist/antagonist of opioid receptors, has been used for postoperative pain control [6–9]. Because of its partial μ-agonism, some common adverse effects of opioids are significantly reduced with this drug [10], indicating that it may be a good alternative for controlling postoperative pain. The goal of this study was to conduct a comprehensive review of randomized controlled trials (RCTs) to assess the analgesic efficacy and safety of dezocine for preventing moderate to severe postoperative pain.

## Methods

This meta-analysis was conducted according to the Cochrane Collaboration [11] and Preferred Reporting Items for Systematic Reviews and Meta-Analyses (PRISMA) Statement guidelines [12].

### Search Strategy

We searched the Medline, EMBASE, and Cochrane Central Register of Controlled Trials (CENTRAL) databases to identify all RCTs that investigated the analgesic efficacy of dezocine on postoperative pain using the strategies shown in the Appendix. Potentially eligible studies were also identified through a manual search of the references and citations in the articles retrieved for full review. The searches were last updated in May 2015.

### Selection

All clinical trials that used a randomized and controlled study design and assessed treatment with dezocine during the perioperative period were included. Two authors initially reviewed the titles and abstracts to exclude irrelevant studies and subsequently screened the potentially eligible studies by a detailed review of the full-text article to determine eligibility. Disagreements were resolved by consensus with a third author.

### Data Extraction

Two authors independently extracted data from the full-text article of each included study using a standardized data collection form (Table 1). The primary outcomes for the current review were the number of patients with at least 50% pain relief and overall evaluations of physician and patient satisfaction. The secondary outcome was adverse events. The analgesic potency of dezocine is considered to be similar to that of morphine [6]. If dezocine and morphine were administered at different dosages in the same study, data on the most reported dose of 10 mg dezocine or 10 mg morphine dose were used. If necessary, authors were contacted to obtain further data.

**Table 1:** Characters of included studies.

Reference	Participant						Intervention			Control drugs	Outcome	
	Number (placebo/dezocine/morphine)	Sex (Female %)	Age (mean, years)	ASA classification	Type of surgery	Pain intensity	Route and time of administration	Doses of dezocine	Other analgesic use	Type and dose	Measurements	Time points
Downing et al. (1981)	40/40	100	NA	NA	lower abdominal surgery.	moderate to severe	i.m. postoperation	dezocine 5, 10, 15mg	NA	Morphine 10mg	patient’s self-rating of pain intensity and pain relies; adverse events	15min, 30min, 1h, 2h, 4h after injection
Edwards et al. (1986)	38/38/41	63	35.4	I-II	various surgical procedures	moderate to severe	i.m. postoperation	dezocine 10, 20mg	NA	placebo and 10mg morphine	patient’s self-rating of pain intensity and pain relies; overall evaluation of physician and patient satisfaction; adverse events	15min, 30min, and hourly up to 6h after injection
Finucane et al. (1986)	37/39	67	32.7	I-II	various surgical procedures	moderate to severe	i.m. postoperation	dezocine 10, 15mg	NA	placebo and 2mg butorphanol	patient’s self-rating of pain intensity and pain relies; overall evaluation of physician and patient satisfaction; adverse events	15min, 30min, and hourly up to 6h after injection
Galloway et al. (1986)	40/41	58	32	I-II	intraabdominal, orthopedic, and general surgical procedures	moderate to severe	i.v. postoperation	dezocine 5, 10mg	NA	placebo and 1mg butorphanol	patient’s self-rating of pain intensity and pain relies; overall evaluation of physician and patient satisfaction; adverse events	15min, 30min, and hourly up to 6h after injection
Gravenstein et al. (1984)	39/39/40	50	43	NA	NA	moderate to severe	i.m. postoperation	dezocine 10, 15mg		placebo and 10mg morphine	patient’s self-rating of pain intensity and pain relies; overall evaluation of physician and patient satisfaction; adverse events	15min, 30min, and hourly up to 6h after injection
Pandit et al. (1985	38/37/39	70	34	I-II	various surgical procedures	moderate to severe	i.m. postoperation	dezocine 5, 10, 15mg	NA	placebo and 10mg morphine	patient’s self-rating of pain intensity and pain relies; overall evaluation of physician satisfaction; adverse events	15min, 30 min, 1h, 2h, 4h after injection
Pandit et al. (1986)	41/38	67	36	I-II	general surgical, gynecologic, and orthopedic procedures	moderate to severe	i.v. postoperation	dezocine 2.5, 5,10 mg	NA	placebo and 5mg morphine	patient’s self-rating of pain intensity and pain relies; overall evaluation of physician and patient satisfaction; adverse events	15min, 30min, and hourly up to 6h after injection

i.m. = intramuscular; i.v. = intravenous; NA = not applicable

### Quality Assessment

A quality assessment was independently performed by two authors using an established tool, the Jadad scale [13], which is widely used to assess the methodological quality of clinical trials. This scale included the method of randomization (2 points), double blinding (2 points), and the description of dropouts (1 point). Disagreements were resolved by consensus with a third author.

### Statistical Analysis

All calculations were performed using RevMan software version 5.2 (Cochrane Collaboration). Dichotomous data were expressed as the relative risk (RR) and 95% confidence intervals (CIs). The data were pooled using a more conservative Mantel-Haenszel random effects model because of anticipated heterogeneity among included studies. Heterogeneity among the studies was tested using the I^2^ statistic with values >50% and Chi^2^ test with P ≤ 0.05 indicating strong heterogeneity between the studies [14]. Sensitivity analyses were performed to evaluate the primary outcomes according to the route of administration and gender or by excluding studies with a high risk of bias. Due to the limited number of studies (<10) included in each analysis, we did not construct funnel plots to assess publication probability [15].

## Results

**Fig 1:**
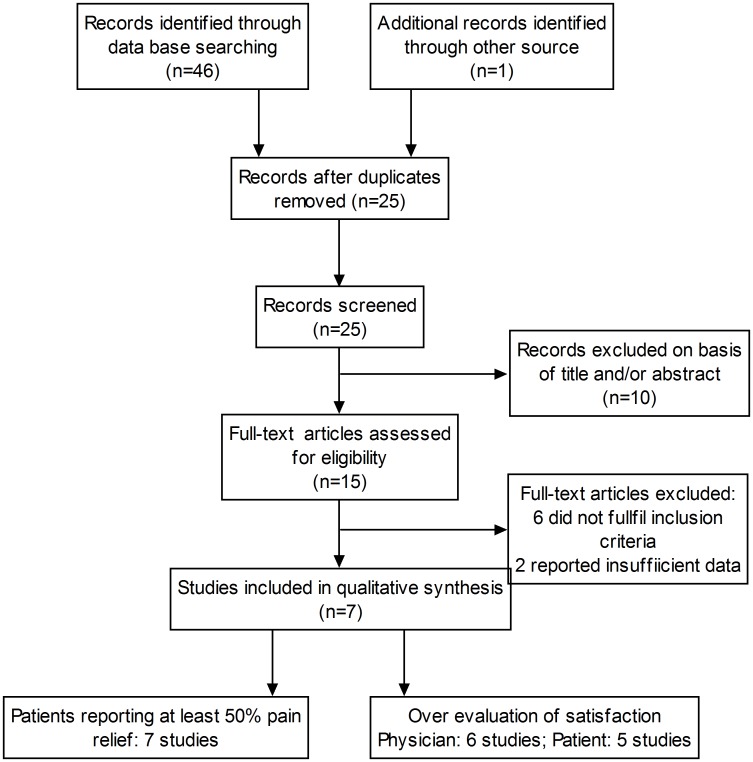
PRISMA flow chart detailing retrieved, excluded, assessed, and included trials.

Our search strategy identified 47 potentially relevant studies. After a number of the studies were excluded, 7 intermediate- to high-quality studies [6,7,16–20] with a total of 665 patients were finally included in this meta-analysis (Fig 1). The characteristics of these studies and the quality scores are shown in Tables 1 and 2.

**Table 2:** Quality analysis of included studies.

Reference	Randomization	Blinding	Describing withdrawals	Allocation concealment	Jadad’s scores
Downing et al. (1981)	randomization tables	double blind	yes	unclear	5B
Edwards (1986)	just mentioned random	double blind	yes	unclear	4B
Finucane (1986)	Unclear	double blind	NA	unclear	2B
Galloway (1986)	just mentioned random	double blind	yes	unclear	4B
Gravenstein et al. (1984)	just mentioned random	double blind	yes	unclear	4B
Pandit (1985)	just mentioned random	double blind	yes	sealed ampoules	4A
Pandit (1986)	just mentioned random	double blind	yes	unclear	4B

### Dezocine versus placebo

#### Number of patients with at least 50% pain relief

**Fig 2:**
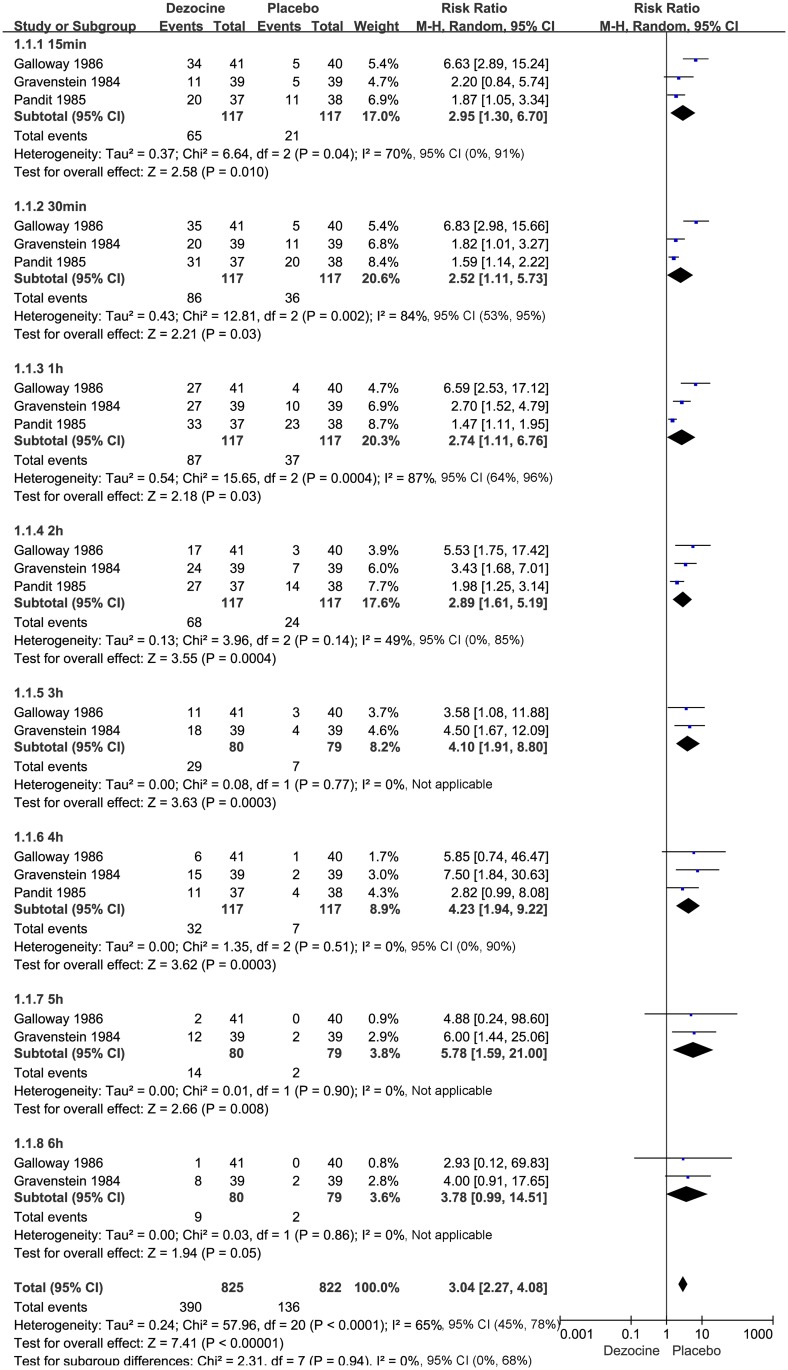
Forest plot of the effect of dezocine versus placebo on postoperative pain relief (at least 50%) within 6 h after surgery. RR = relative risk; and CI = confidence interval.

Of the 7 included studies, 3 [7,18,20] (n = 234) compared the effectiveness of dezocine versus placebo according to the number of patients with at least 50% pain relief achieved within 0–6 h after surgery. These three studies found that dezocine significantly increased the proportion of patients with at least 50% pain relief within 0–6 h after surgery (15 min: N = 234; RR 2.95, 95% CI 1.30 to 6.70; I² = 70%, 95% CI 0% to 91%; χ^2^ = 6.64, P = 0.004; 30 min: N = 234; RR 2.52, 95% CI 1.11 to 5.73; I² = 84%, 95% CI 53% to 95%; χ^2^ = 12.81, P = 0.002; 1 h: N = 234; RR 2.74, 95% CI 1.11 to 6.76; I² = 87%, 95% CI 64% to 96%; χ^2^ = 15.65, P = 0.0004; 2 h: N = 234; RR 2.89, 95% CI 1.61 to 5.19; I² = 49%, 95% CI 0% to 85%; χ^2^ = 3.96, P = 0.14; 3 h: N = 159; RR 4.10, 95% CI 1.91 to 8.80; I² = 0%, 95% CI is not applicable; χ^2^ = 0.08, P = 0.77; 4 h: N = 234; RR 4.23, 95% CI 1.94 to 9.22; I² = 0%, 95% CI 0% to 90%; χ^2^ = 1.35, P = 0.51; 5 h: N = 159, RR 5.78, 95% CI 1.59 to 21.00; I² = 0%, 95% CI is not applicable; χ^2^ = 0.01, P = 0.90; and 6 h: N = 159; RR 3.78, 95% CI 0.99 to 14.51; I² = 0%, 95% CI is not applicable; χ^2^ = 0.03, P = 0.86). The results are summarized in the forest plot in Fig 2.

#### Physician satisfaction

**Fig 3:**
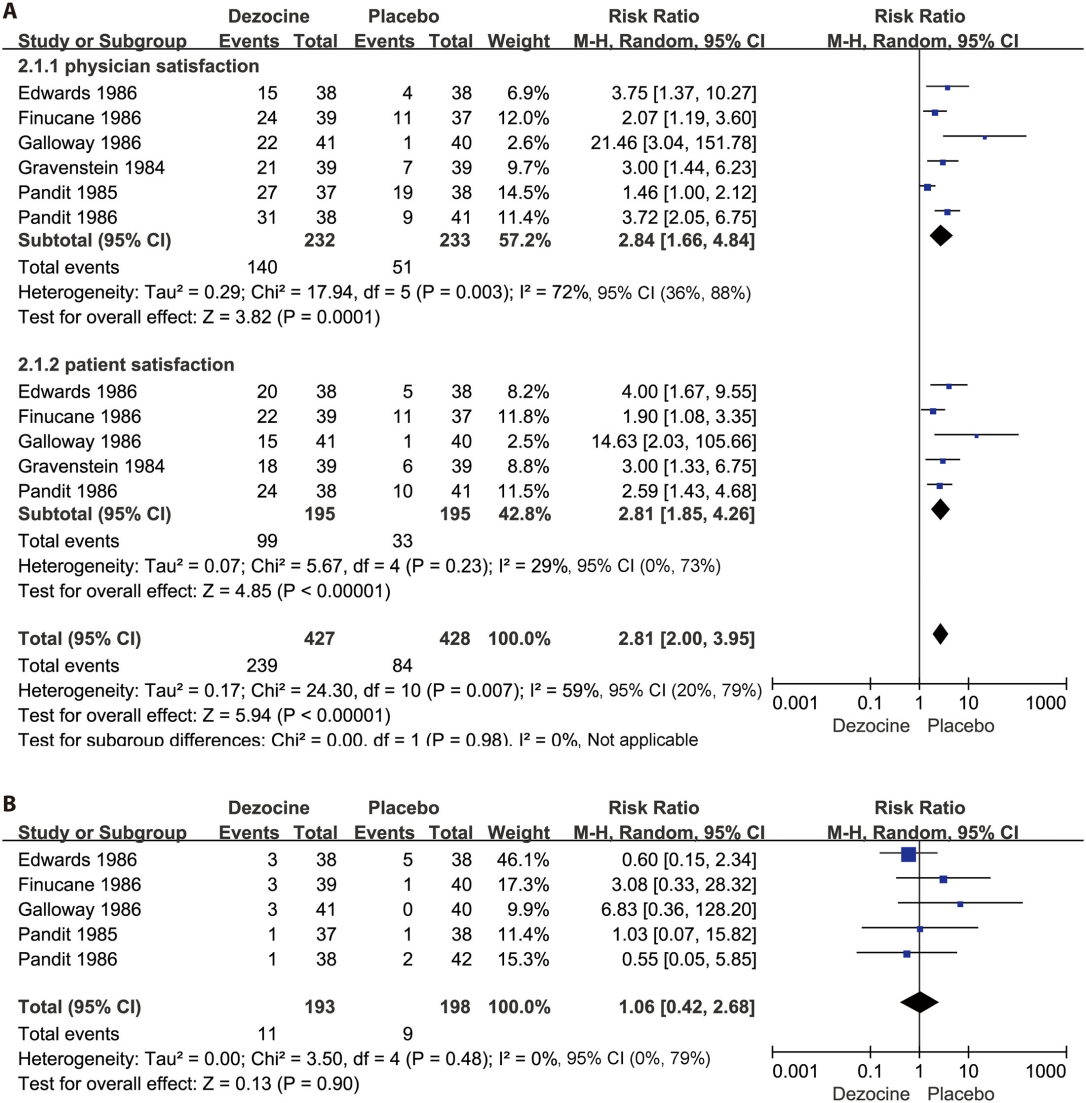
Forest plot of the effects of dezocine versus placebo on physician and patient satisfaction after surgery (A), and postoperative nausea and vomiting (PONV) (B). RR = relative risk; and CI = confidence interval.

Of the included studies, six [7,16–20] (n = 465) compared the effects of dezocine versus placebo on physician satisfaction. These six studies showed that dezocine significantly increased the overall degree of physician satisfaction compared with the placebo (N = 465; RR 2.84, 95% CI 1.66 to 4.84; I² = 72%, 95% CI 36% to 88%; χ^2^ = 17.94, P = 0.003). The physician satisfaction results are presented in the forest plot in Fig 3A.

#### Patient satisfaction

Five studies [7,16,17,19,20] (n = 390) compared the effectiveness of dezocine versus placebo on patient satisfaction. These five trials showed that dezocine significantly increased the overall degree of patient satisfaction compared with the placebo (N = 390; RR 2.81, 95% CI 1.85 to 4.26; I² = 29%, 95% CI 0% to 73%; χ^2^ = 5.67, P = 0.23). The patient satisfaction results are presented in the forest plot in Fig 3A.

#### Adverse events

The most commonly reported adverse event in the included studies [7,16–19] was postoperative nausea and vomiting (PONV). However, the incidence of PONV was similar between the dezocine and placebo groups (N = 291; RR 1.06, 95% CI 0.42 to 2.68; I² = 0%, 95% CI 0% to 79%; χ^2^ = 3.50, P = 0.48). The pooled RRs for PONV are presented in the forest plot in Fig 3B.

### Dezocine versus Morphine

#### Number of patients with at least 50% pain relief

**Fig 4:**
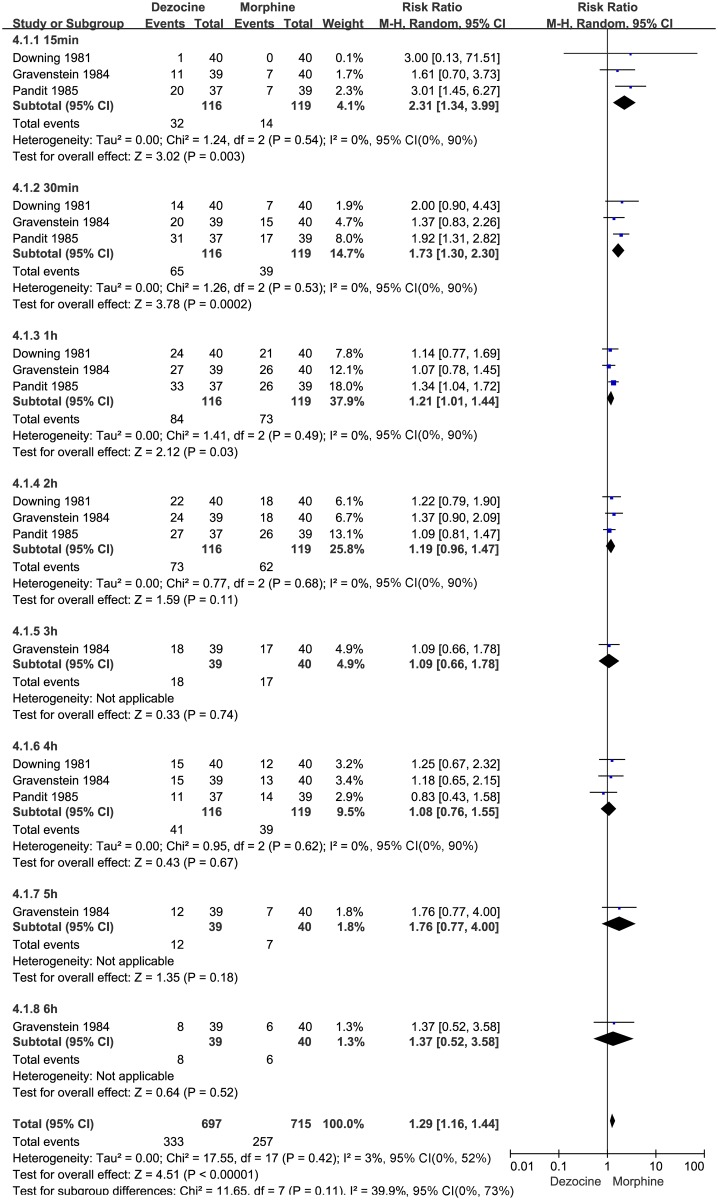
Forest plot of the effect of dezocine versus morphine on the overall evaluation of physician satisfaction after surgery. RR = relative risk; and CI = confidence interval.

Three studies [6,18,20] (n = 235) compared the effectiveness of dezocine versus morphine according to the number of patients reporting at least 50% pain relief. These three studies showed that dezocine had an effect similar to morphine, as demonstrated by the similar numbers of patients with at least 50% pain relief reported during the late postoperative period (within 2–6 h) (2 h: N = 235; RR 1.19, 95% CI 0.96 to 1.47; I² = 0%, 95% CI 53% to 95%; χ^2^ = 0.77, P = 0.68; 3 h: N = 79, RR 1.09, 95% CI 0.66 to 1.78; 4 h: N = 235, RR 1.08, 95% CI 0.76 to 1.55; I² = 0%, 95% CI 0% to 90%; χ^2^ = 0.95, P = 0.62; 5 h: N = 79, RR 1.76, 95% CI 0.77 to 4.00; and 6 h: N = 79, RR 1.37, 95% CI 0.52 to 3.58). However, dezocine caused a significant increase in the number of patients with at least 50% pain relief during the early postoperative period (within 0-1h) compared with morphine (15 min: N = 235; RR 2.31, 95% CI 1.34 to 3.99; I² = 0%, 95% CI 0% to 90%; χ^2^ = 1.24, P = 0.54; 30 min: N = 235; RR 1.73, 95% CI 1.30 to 2.30; I² = 0%, 95% CI 0% to 90%; χ^2^ = 1.26, P = 0.53; and 1 h: N = 235; RR 1.21, 95% CI 1.01 to 1.44; I² = 0%, 95% CI 0% to 90%; χ^2^ = 1.41, P = 0.49). The results are summarized in the forest plot in Fig 4.

#### Physician satisfaction

**Fig 5:**
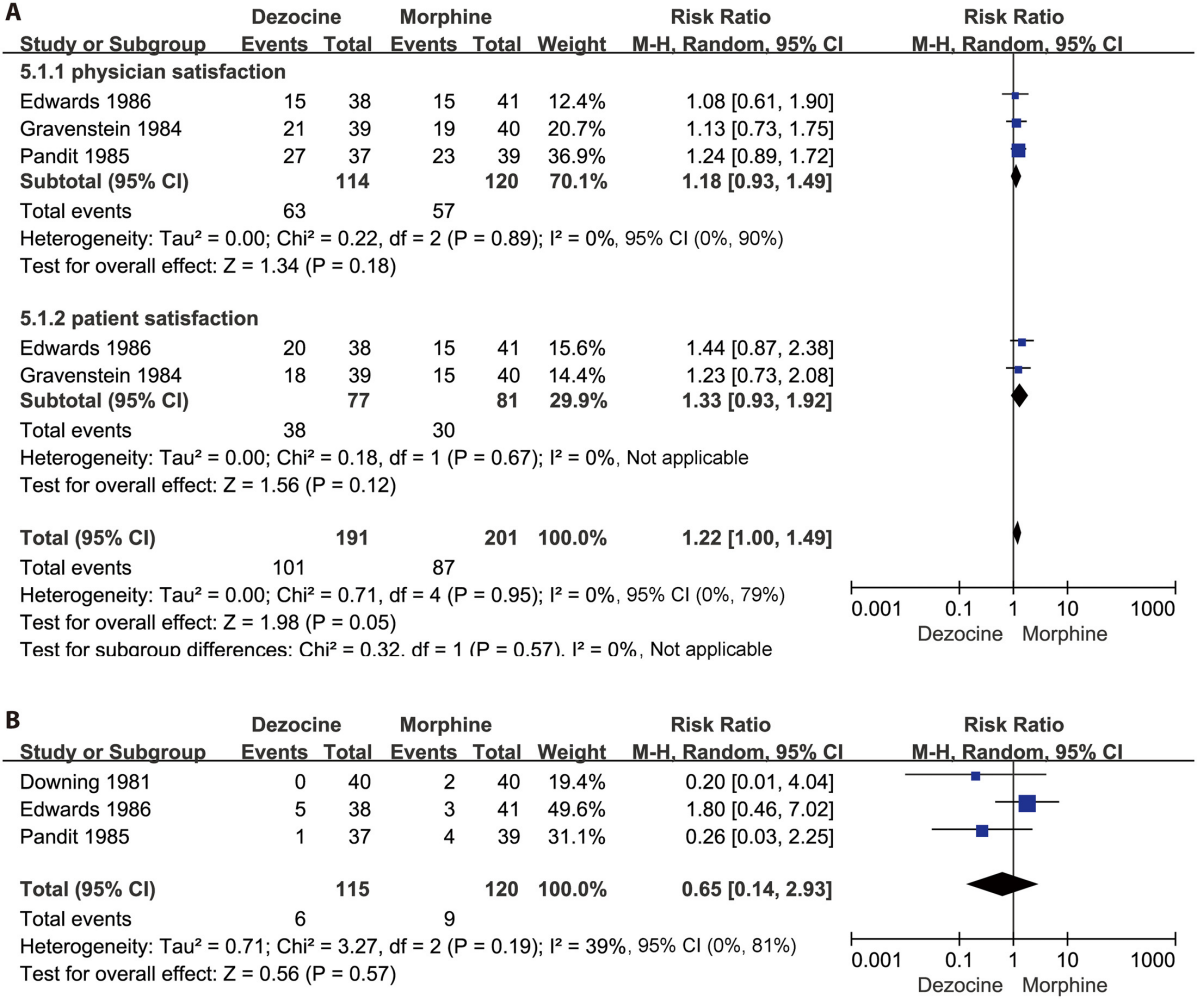
Forest plot of the effects of dezocine versus morphine on physician and patient satisfaction after surgery (A), and postoperative nausea and vomiting (PONV) (B). RR = relative risk; and CI = confidence interval.

Three studies [16,18,20] (n = 234) compared the effectiveness of dezocine versus morphine on physician satisfaction. These three studies showed that dezocine did not have a significant effect on physician satisfaction compared with morphine (N = 234; RR 1.18, 95% CI 0.93 to 1.49; I² = 0%, 95% CI 0% to 90%; χ^2^ = 0.22, P = 0.89). The results are presented in the forest plot in Fig 5A.

#### Patient satisfaction

Two studies [16,20] (n = 158) compared the effect of dezocine versus morphine on patient satisfaction. These two studies showed that dezocine did not have a significant effect on increasing patient satisfaction (N = 158; RR 1.33, 95% CI 0.93 to 1.92; I² = 0%, 95% CI is not applicable; χ^2^ = 0.71, P = 0.95) compared with morphine. The results of patient satisfaction between dezocine and morphine are presented in the forest plot in Fig 5A.

#### Adverse events

Three studies [6,16,18] (n = 235) compared PONV with dezocine or morphine. Analysis showed that dezocine had no significant advantage over morphine in terms of PONV (N = 235; RR 0.65, 95% CI 0.14 to 2.93; I² = 39%, 95% CI 0% to 81%; χ^2^ = 3.27, P = 0.19). The pooled RR values for PONV are presented in the forest plot in Fig 5B.

### Sensitivity Analysis

Restricting analysis to studies in which dezocine was only administered intramuscularly or intravenously or those only involving female patients did not change the directions of the primary outcomes. Following the exclusion of one study with a high risk bias [17], dezocine still resulted in significant increases in physician and patient satisfaction compared with the placebo.

All the meta-analysis results are summarized in Table 3.

**Table 3:** Summary of outcomes.

Outcome measures	Time	Number of studies	Number of patients	Pooled RR (random effect model)			Heterogeneity			
				RR (95%CI)	z	p	χ^2^	P	I^2^ (%, 95% CI)	Tau^2^
**Dezocine versus Placebo**										
Number of patients with at least 50% pain relief	15min	3^7,18,20^	234	2.95 (1.30, 6.70)	2.58	0.01	6.64	0.04	0.70 (0.00, 0.91)	0.37
	30min	3^7,18,20^	234	2.52 (1.11, 5.73)	2.21	0.03	12.81	0.002	0.84 (0.53, 0.95)	0.43
	1h	3^7,18,20^	234	2.74 (1.11, 6.76)	2.18	0.03	15.65	0.0004	0.87 (0.64, 0.96)	0.54
	2h	3^7,18,20^	234	2.89 (1.61, 5.19)	3.55	0.0004	3.96	0.14	0.49 (0.00, 0.85)	0.13
	3h	2^7,20^	159	4.10 (1.91, 8.80)	3.63	0.0003	0.08	0.77	0.00 (NA)	0.00
	4h	3^7,18,20^	234	4.23 (1.94, 9.22)	3.62	0.0003	1.35	0.51	0.00 (0.00, 0.90)	0.00
	5h	2^7,20^	159	5.78 (1.59, 21.00)	2.66	0.008	0.01	0.90	0.00 (NA)	0.00
	6h	2^7,20^	159	3.78 (0.99, 14.51)	1.94	0.05	0.03	0.86	0.00 (NA)	0.00
Physician satisfaction	6h	6^7,16,17,18,19,20^	465	2.84 (1.66, 4.84)	3.82	0.0001	17.94	0.003	0.72 (0.36, 0.88)	0.29
Patient satisfaction	6h	5^7,16,17,19,20^	390	2.81 (1.85, 4.26)	4.85	<0.00001	5.67	0.23	0.29 (0.00, 0.73)	0.07
Postoperative nausea and vomiting (PONV)	6h	5^7,16,17,18,19^	391	1.06 (0.42, 2.68)	0.13	0.90	3.50	0.48	0.00 (0.00, 0.79)	0.00
**Dezocine versus Morphine**										
Number of patients with at least 50% pain relief	15min	3^6,18,20^	235	2.31 (1.34, 3.99)	3.02	0.003	1.24	0.54	0.00 (0.00, 0.90)	0.00
	30min	3^6,18,20^	235	1.73 (1.30, 2.30)	3.78	0.0002	1.26	0.53	0.00 (0.00, 0.90)	0.00
	1h	3^6,18,20^	235	1.21 (1.01, 1.44)	2.12	0.03	1.41	0.49	0.00 (0.00, 0.90)	0.00
	2h	3^6,18,20^	235	1.19 (0.96, 1.47)	1.59	0.11	0.77	0.68	0.00 (0.00, 0.90)	0.00
	3h	1^20^	79	1.09 (0.66, 1.78)	0.33	0.74	NA	NA	NA	NA
	4h	3^6,18,20^	235	1.08 (0.76, 1.55)	0.43	0.67	0.95	0.62	0.00 (0.00, 0.90)	0.00
	5h	1^20^	79	1.76 (0.77, 4.00)	1.35	0.18	NA	NA	NA	NA
	6h	1^20^	79	1.37 (0.52, 3.58)	0.64	0.52	NA	NA	NA	NA
Physician satisfaction	6h	3^16,18,20^	234	1.18 (0.93, 1.49)	1.34	0.18	0.22	0.89	0.00 (0.00, 0.90)	0.00
Patient satisfaction	6h	2^16,20^	158	1.33 (0.93, 1.92)	1.56	0.12	0.18	0.67	NA	0.00
Postoperative nausea and vomiting (PONV)	6h	3^6,16,18^	235	0.65 (0.14, 2.93)	0.56	0.57	3.27	0.19	0.39 (0.00, 0.81)	0.71

## Discussion

This is the first meta-analysis to assess the analgesic effects of dezocine in preventing postoperative pain. Our results suggested that the administration of this drug was indeed effective in this regard.

Dezocine, a mixed agonist/antagonist of opioid receptors, was first synthesized in the 1970s [21]. Although its specific mechanism of action is still not completely understood [22], there is no doubt that it has potent analgesic effects. As early as 1978, Fragen RJ and Caldwell have reported that dezocine can be used as a postoperative pain-killing drug [21]. Intramuscular dezocine has also been used to manage chronic cancer pain, and its analgesic effects are superior to those of butorphanol [23]. Furthermore, in a neuropathic pain mouse model, researchers found that dezocine significantly attenuated peripheral nerve injury-induced thermal and mechanical pain hyperalgesia [24]. Although, these results suggest the potential use of dezocine as an alternative medication for the treatment of pain, the effect of dezocine on postoperative pain have mostly been investigated in studies with small sample sizes, low statistical power, or other limitations in study design, leading to conflicting results. In the present study, we performed a meta-analysis using all available related studies to better define the efficacy and safety of dezocine, and found that it was effective in reducing postoperative pain.

Of the trials that reported dezocine-related adverse events, there was no difference between groups in the incidence of PONV. Only one study [7] assessed the effect of dezocine on respiratory depression, however, no patient showed signs of clinically significant respiratory depression. It was impossible to quantitatively assess the effect of dezocine on drug addiction because a number of the trials were short-term (within 6 h after surgery) and involved the administration of only a single dose. The conclusion that dezocine is a safe alternative medication for postoperative pain should be drawn with caution because of the low incidence of side effects reported and the limited number of small-scale trials included in this study.

The analgesic potency of dezocine has generally been considered to be similar to that of morphine. In this review, we found that dezocine did not cause a significantly different effect on postoperative pain relief within 2–6 h after surgery or on physician or patient satisfaction compared with the same dose of morphine. However, we did find significantly better pain relief associated with the use of dezocine within the first hour after surgery. These results suggest that the analgesic effect of dezocine is equal to but may act faster than morphine. The use of morphine to prevent postoperative pain can cause opioid-related adverse events, such as PONV. However, we did not detect a significant difference in PONV following dezocine compared with morphine administration in our analysis. It is likely that the limited number of small-scale trials included in the current review reporting low incidences of PONV contributed to this result.

Certainly, this review has some limitations. First, the limited number of small-scale trials included in this review prevents solid conclusions from being drawn. Second, we combined different types of surgery, both genders, and different routes of administration in our main analyses, which created heterogeneity in many of the results. Due to the small number of trials, we did not perform subgroup or meta-regression analysis to assess the impact of these factors on the main outcomes. The results from these analyses may be biased and must be interpreted with caution. In addition, we have quite a few zero estimates on heterogeneity. However, the small number of trials could also made a false assumption on these weak heterogeneities. It is very likely to not be picking existing heterogeneity or be underestimating them [25]. Third, most trials in the current study were conducted before 1990, and the presentation of the data was unsatisfactory. We found that only dichotomous data, such as the number of patients reporting at least 50% pain relief, were useful for performing meta-analysis. Individual patient data (IPD) meta-analyses might be useful to solve these problems. However, because the studies are old, it is unlikely anyone would be able to get hold of the original datasets. In addition, recent evidence suggests that publication bias is much more likely in older studies, as expected, which implies that bias might be present in this meta-analysis.

Some areas for future research have been identified. Pain relief was achieved in only seven of the small-sample trials. Large, adequately powered studies are needed to compare the efficacy and adverse effects of dezocine. In addition, further investigation is needed to assess the optimal dose, alternative modes of administration, and time points of dezocine dosing for reducing postoperative pain. Studies assessing the differential analgesic effects of dezocine and morphine are also needed.

In summary, the administration of dezocine is associated with increases in the number of patients reporting at least 50% pain relief as well as physician and patient satisfaction. Additional large studies will be necessary to adequately evaluate the side effects of this drug.

## Supporting Information

S1 Appendix Search Strategy. (DOCX)

S1 File Review Manager file. (ZIP)

S1 Table PRISMA checklist. (DOC)
