# Supplementary material for: Dezocine for Preventing Postoperative Pain: A Meta-Analysis of Randomized Controlled Trials
Source: PLoS One. 2015 Aug 19;10(8):e0136091. doi: 10.1371/journal.pone.0136091 (PMC4545891; doi:10.1371/journal.pone.0136091)
Supplement: S1 Appendix — (DOCX) [file pone.0136091.s001.docx]

**Appendix 1. MEDLINE via Ovid search strategy**

1. Dezocine.sh.

2. ((WY 16225) or WY-16225).ti.ab.kw.

3. dalgan.ti.ab.kw.

4. OR/1-3

5. Pain, Postoperative.sh.

6. ((postoperative adj4 pain$) or (post-operative adj4 pain$) or post-operative-pain$ or (post$ NEAR pain$) or (postoperative adj4 analgesi$) or (post-operative adj4 analgesi$) or (“post-operative analgesi$”)).ti.ab.kw.

7. ((post-surgical adj4 pain$) or (“post surgical” adj4 pain$) or (post-surgery adj4 pain$)).ti.ab.kw.

8. ((“pain-relief after surg$”) or (“pain following surg$”) or (“pain control after”)).ti.ab.kw.

9. ((“post surg$” or post-surg$) AND (pain$ or discomfort)).ti.ab.kw.

10. ((pain$ adj4 “after surg$”) or (pain$ adj4 “after operat$”) or (pain$ adj4 “follow$ operat$”) or (pain$ adj4 “follow$ surg$”)).ti.ab.kw.

11. ((analgesi$ adj4 “after surg$”) or (analgesi$ adj4 “after operat$”) or (analgesi$ adj4 “follow$ operat$”) or (analgesi$ adj4 “follow$ surg$”)).ti.ab.kw.

12. OR/5-11

13. randomized controlled trial.pt.

14. controlled clinical trial.pt.

15. randomized.ab.

16. placebo.ab.

17. drug therapy.fs.

18. randomly.ab.

19. trial.ab.

20. groups.ab.

21. OR/13-20

22. humans.sh.

23. 21 AND 22

24. 4 AND 12 AND 23

**Appendix 2. Search strategy for EMBASE via Ovid**

1. Dezocine.sh.

2. ((WY 16225) or WY-16225).ti.ab.kw.

3. dalgan.ti.ab.kw.

4. OR/1-3

5. Postoperative pain.sh.

6. ((postoperative adj4 pain$) or (post-operative adj4 pain$) or post-operative-pain$ or (post$ NEAR pain$) or (postoperative adj4 analgesi$) or (post-operative adj4 analgesi$) or (“post-operative analgesi$”)).ti.ab.kw.

7. ((post-surgical adj4 pain$) or (“post surgical” adj4 pain$) or (post-surgery adj4 pain$)).ti.ab.kw.

8. ((“pain-relief after surg$”) or (“pain following surg$”) or (“pain control after”)).ti.ab.kw.

9. ((“post surg$” or post-surg$) AND (pain$ or discomfort)).ti.ab.kw.

10. ((pain$ adj4 “after surg$”) or (pain$ adj4 “after operat$”) or (pain$ adj4 “follow$ operat$”) or (pain$ adj4 “follow$ surg$”)).ti.ab.kw.

11. ((analgesi$ adj4 “after surg$”) or (analgesi$ adj4 “after operat$”) or (analgesi$ adj4 “follow$ operat$”) or (analgesi$ adj4 “follow$ surg$”)).ti.ab.kw.

12. OR/5-11

13. Clinical trials.sh.

14. Controlled Clinical Trials.sh.

15. Randomized Controlled Trial.sh.

16. Double-blind procedure.sh.

17. (clin$ adj25 trial$).ab.

18. ((doubl$ or trebl$ or tripl$) adj25 (blind$ or mask$)).ab.

19. placebo$.ab.

20. random$.ab.

21. OR/13-20

22. 4 AND 12 AND 21

**Appendix 3. Search strategy for Cochrane CENTRAL**

1. MESH descriptor Dezocine

2. (WY 16225) or WY-16225):ti,ab,kw.

3. dalgan.ti.ab.kw.

4. OR/1-3

5. MESH descriptor Pain, Postoperative

6. ((postoperative adj4 pain$) or (post-operative adj4 pain$) or post-operative-pain$ or (post$ NEAR pain$) or (postoperative adj4 analgesi$) or (post-operative adj4 analgesi$) or (“post-operative analgesi$”)):ti,ab,kw.

7. ((post-surgical adj4 pain$) or (“post surgical” adj4 pain$) or (post-surgery adj4 pain$)):ti,ab,kw.

8. ((“pain-relief after surg$”) or (“pain following surg$”) or (“pain control after”)):ti,ab,kw.

9. ((“post surg$” or post-surg$) AND (pain$ or discomfort)):ti,ab,kw.

10. ((pain$ adj4 “after surg$”) or (pain$ adj4 “after operat$”) or (pain$ adj4 “follow$ operat$”) or (pain$ adj4 “follow$ surg$”)):ti,ab,kw.

11. ((analgesi$ adj4 “after surg$”) or (analgesi$ adj4 “after operat$”) or (analgesi$ adj4 “follow$ operat$”) or (analgesi$ adj4 “follow$ surg$”)):ti,ab,kw.

12. OR/4-10

13. Limit 11 to Clinical Trials (CENTRAL)
